# A comparison of dose-response characteristics of four NTCP models using outcomes of radiation-induced optic neuropathy and retinopathy

**DOI:** 10.1186/1748-717X-6-61

**Published:** 2011-06-06

**Authors:** Vitali Moiseenko, William Y Song, Loren K Mell, Niranjan Bhandare

**Affiliations:** 1 British Columbia Cancer Agency, Vancouver Cancer Centre, 600 W. 10th Ave, Vancouver, BC, V5Z 4E6, Canada; 2 University of California San Diego, Rebecca and John Moores Comprehensive Cancer Center, 3855 Health Sciences Drive, La Jolla, CA, 92093-0843, USA; 3 University of Florida Health Sciences Center, P.O. Box 100385, Gainesville, FL, 32610-0385, USA

## Abstract

**Background:**

Biological models are used to relate the outcome of radiation therapy to dose distribution. As use of biological models in treatment planning expands, uncertainties associated with the use of specific models for predicting outcomes should be understood and quantified. In particular, the question to what extent model predictions are data-driven or dependent on the choice of the model has to be explored.

**Methods:**

Four dose-response models--logistic, log-logistic, Poisson-based and probit--were tested for their ability and consistency in describing dose-response data for radiation-induced optic neuropathy (RION) and retinopathy (RIRP). Dose to the optic nerves was specified as the minimum dose, *D_min_*, received by any segment of the organ to which the damage was diagnosed by ophthalmologic evaluation. For retinopathy, the dose to the retina was specified as the highest isodose covering at least 1/3 of the retinal surface (*D_33%_*) that geometrically covered the observed retinal damage. Data on both complications were modeled separately for patients treated once daily and twice daily. Model parameters *D_50 _*and *γ *and corresponding confidence intervals were obtained using maximum-likelihood method.

**Results:**

Model parameters were reasonably consistent for RION data for patients treated once daily, *D_50 _*ranging from 94.2 to 104.7 Gy and *γ *from 0.88 to 1.41. Similar consistency was seen for RIRP data which span a broad range of complication incidence, with *D_50 _*from 72.2 to 75.0 Gy and *γ *from 1.51 to 2.16 for patients treated twice daily; 72.2-74.0 Gy and 0.84-1.20 for patients treated once daily. However, large variations were observed for RION in patients treated twice daily, D_50 _from 96.3 to 125.2 Gy and *γ *from 0.80 to 1.56. Complication incidence in this dataset in any dose group did not exceed 20%.

**Conclusions:**

For the considered data sets, the log-logistic model tends to lead to larger *D_50 _*and lower *γ *compared to other models for all datasets. Statements regarding normal tissue radiosensitivity and steepness of dose-response, based on model parameters, should be made with caution as the latter are not only model-dependent but also sensitive to the range of complication incidence exhibited by clinical data.

## Background

Modeling of dose-volume response for normal tissues has been used to establish correlation between toxicity and dose-volume parameters, determine safe dose distributions in organs at risk and make projections for risks of adverse effects associated with dose escalation. Biologically-based radiotherapy optimization has progressed in recent years from pioneering work presenting the concept [1-3] to commercial implementation [4]. It is expected that biologically-based radiotherapy planning will play a more prominent role. This could be facilitated by expanding use of biological imaging intended to map biological properties of tumors and organs at risk [5,6] thereby making planning not only biologically-based but also patient-specific [7].

The dose-response follows the basic sigmoid shape and numerous models have been proposed based either on a purely statistical approach or assumptions regarding organ architecture and its influence on the development of complications [8]. The popular choices to describe the sigmoid dose-response curves are: Poisson-based, probit, logistic and log-logistic functions [9-12]. Dose-response can be plotted as a function of a dosimetric parameter deemed significant for a particular complication. This can be mean or maximum dose or equivalent uniform dose (also known as effective dose), EUD [13]. If the intent of the model is to specifically account for volume effect, typically a parameter to account for this effect is introduced [9,10]. Fits to multiple models have been reported in the literature [14,15]. The purpose of these studies is typically two-fold: 1) to establish a model that provides the most accurate description of clinical data and; 2) to test consistency of model predictions, e.g., strength of volume effects.

**Figure 1:**
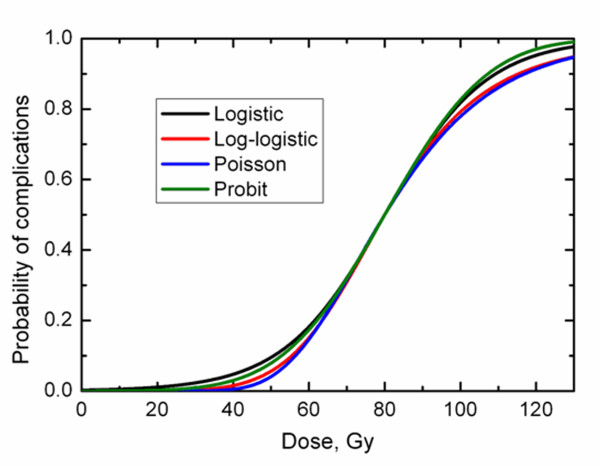
**Dose-response predicted by four studied models**. Model parameters were commonly set to D_50 _= 80 Gy and γ = 1.5.

A sigmoid curve can be readily described by a two-parameter function, one parameter describing the dose at which 50% of patients exhibit complications, *D_50_*, and the second parameter, *γ*, the normalized dose-response gradient [16]. Because all models follow a similar sigmoid shape it is generally acknowledged that fits to typically noisy human data do not allow establishing superiority of a particular model over other models [8]. It is further acknowledged that different models with the same *D_50 _*and *γ *would follow a similar dose-response. Figure 1 shows the dose - response relationship predicted by the four above-mentioned models with matching *D_50 _*= 80 Gy and *γ *= 1.5. The curves overlap around 50% incidence but separate in the low- and high-dose regions. It is, therefore, also acknowledged that model parameters are not interchangeable. That is, *D_50 _*and *γ *obtained following the fitting of one model to a specific data set should not be used with another model. (Figure 1)

Bentzen and Tucker, 1997, provided the most detailed and insightful analysis of specific features of the Poisson-based, logistic and probit models. The authors carefully considered the location of the maximum dose-response slope and maximum normalized dose-response gradient for these models and relationships between measures describing the slope at various response levels. Notably, Bentzen and Tucker, 1997 demonstrated that if logistic and Poisson models are forced to predict identical *D_10_*, dose corresponding to 10% response, and their slopes are matched at *D_10_*, a substantial deviation in *D_50 _*would be observed. Two clinical examples of fitting these three models to describe tumor control probability (TCP) data showed minor variations in *D_50 _*and *γ*. The data used in their clinical example covered a broad range of local control including data points corresponding to 50% TCP.

The emphasis of this report is on normal tissue complications, incidence of which is kept low. This often leaves the parameter *D_50 _*lying outside of the range of clinical data. Despite the stipulations regarding non-transferability of model parameters and ambiguities in quantifying dose-response slope uncovered by Bentzen and Tucker, 1997, the following statements or observations are often made in the literature: 1) organs are classified as radiosensitive or radioresistant based on *D_50_*; 2) dose-response is described as shallow or steep based on *γ*; 3) review articles interpret differences in *D_50 _*and *γ *reported by various institutions as a reflection of differences in underlying data. This is based on an assumption that the parameters governing the dose-response would be reasonably consistent if fitting was performed to the same data set.

Plotting or tabulating model parameters from different studies is a good way to obtain a broad overview of dose-response data. A recently published special issue of the International Journal of Radiation Oncology Biology Physics was dedicated to the Quantitative Analysis of Normal Tissue Effects in the Clinic (QUANTEC). This included 16 consistently structured organ-specific papers [17] and a number of papers contained summarized dose-response parameters in a form of a table or a graph, typically showing a significant spread in these parameters. These comparisons are usually presented in a guarded manner. For example, in the QUANTEC paper on salivary function [18], the plot showing *D_50 _*values for incidence of xerostomia is followed by a qualifying statement that "The wide variation in the reported TD50 values is unexplained but could result from several factors, including differences in dose distributions, salivary measurement methods, segmentation, intragland sensitivity, and so forth". It is, however, notable that three particularly large values of *D_50 _*[19,20] are associated with the use of the log-logistic model, whereas the probit model was used in other studies. Therefore, any systematic and predictable trends and biases in models should be determined and quantified. As will be shown in this work, for the considered data sets, the log-logistic model indeed tends to lead to larger *D_50_*.

Use of model predictions for doses beyond those used in fitting is associated with uncertainties. Marks *et al*. 2010 in their general QUANTEC paper preceding organ-specific QUANTEC articles stipulated: "Some studies use models to estimate the complication risk. Care should be taken when applying models, especially when clinical dose/volume parameters are beyond the range of data". Making projections is, however, one of the purposes of the biological models. These projections are used for a variety of purposes such as changing doses per fraction or dose escalation. Use of model predictions in the dose range not covered by clinical data is unavoidable in IMRT optimization which allows large dose heterogeneity in target volumes and organs at risk which can afford hot spots. Because partial volume response is mathematically connected to NTCP for the whole organ [8], calculating NTCP values for doses on the order of prescription doses is required. As above, any systematic trends and biases should be accounted for. Putting it simply, the question to what extent this is model dependent as well as data-dependent needs to be answered, in particular for severe morbidity incidences which should be kept to manageable minimum.

In this article we present results of fitting radiation-induced optic neuropathy and retinopathy dose-response data to the aforementioned four NTCP models. This is the simplest case where volume dependence is not accounted for and all models have exactly two parameters. Consistency of model parameters, consequences of extrapolating model predictions beyond the dose range covered by clinical data and their dependence on incidence range are reported.

## Methods

### Patient data

Previously reported results of incidence of optic neuropathy and retinopathy in patients treated with radiation for head and neck cancers were used [21,22]. A detailed description of the patient cohort is beyond the scope of this paper. In brief, clinical outcomes data from head and neck cancer patients who received radiation therapy between 1964 and 2000 at the University of Florida were used. Overall incidence of optic neuropathy was 5 in 101 patients treated twice-daily and 19 in 172 patients treated once daily. For retinopathy this incidence was 7 in 78 for patients treated twice daily and 23 in 108 for patients treated once daily. To analyze dose-response for optic neuropathy the dose to the optic nerves was specified as the minimum dose, *D_min_*, received by any segment of the organ to which the damage was diagnosed by ophthalmologic evaluation. For retinopathy the dose to the retina was specified as the highest isodose covering at least 1/3 of the retinal surface (*D_33%_*) that geometrically covered the observed retinal damage. Note that *D_min _*and *D_33% _*apply to segments where damage was seen rather than whole organ. For the purpose of dose-response analysis, dose was converted into normalized total dose (NTD), i.e., isoeffective dose given in 2 Gy fractions. Conversion to NTD was performed using previously reported α/β ratios, 1.76 Gy for optic neuropathy and 2.65 Gy for retinopathy [23,24]. The purpose of this conversion is to aid ease of comparison with literature data. In the remainder of this report terms dose and NTD are used interchangeably, i.e., 2 Gy per fraction is assumed. To test for sensitivity of the model, parameters to α/β value fitting were repeated for the optic neuropathy data set with conversion to NTD performed using α/β values of 1 and 5 Gy.

### NTCP models

Four models were used in this study. Specifically logistic, log-logistic, Poisson-based and probit [9-12] models, equations (1)-(4), respectively.(1)(2)(3)(4)

where *NTCP *is normal tissue complication probability, *D *is dose. For convenience and clarity of presentation the parameter *m *describing the steepness of dose-response in the probit model was converted to common with other models' normalized slope, *γ = D∂NTCP/∂D *using the conversion *γ = [m√(2π)]^-1^*. The formulation of the Poisson-based model shown in equation (3) was proposed by the Stockholm group [9]. A normalized slope for the Poisson-based model maximizes just above NTCP = 1/*e*≈0.37 [9,16]. In contrast, it maximizes at *D_50 _*(log-logistic) or just above *D_50 _*(probit) for other models. The above formulation of the Poisson-based model was criticized because the parameter *γ *never truly equals *D∂NTCP/∂D*, although the difference is small except for very shallow dose-response [16]. At *D_50 _*the normalized slope is equal to *γeln(2)/2*≈0.94*γ*. These inaccuracies were deemed minor for the purposes of this study.

Fitting for *D_50 _*and *γ *was performed using the maximum likelihood method in which parameter values were found that maximized the log-likelihood of the model, given the observed data [25]. The 95% confidence intervals were obtained using the profile likelihood method [26]. Although fitting used individual data points, the figures grouped patient doses in bins of width no larger than 5 Gy. Standard deviations for dose in each group were calculated. Binomial confidence intervals for the incidence of complications were calculated using the score method [27].

## Results

**Figure 2:**
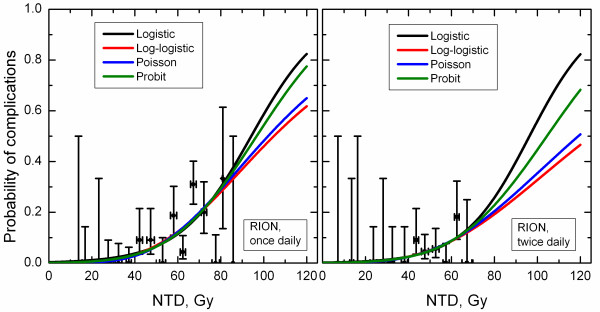
**Incidence of radiation-induced optic neuropathy and dose-response curves predicted by studied four models**. Horizontal error bars show standard deviation for dose for patients from each dose group, vertical error bars are 68% confidence intervals.

**Figure 3:**
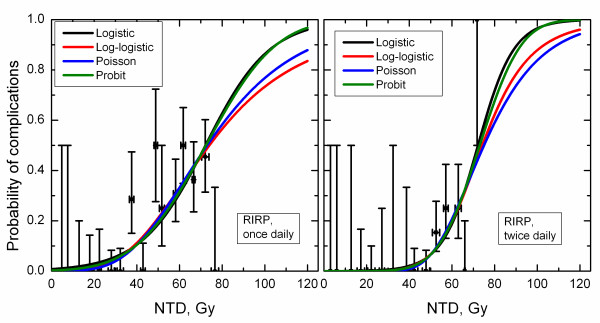
**Incidence of radiation-induced retinopathy and dose-response curves predicted by studied four models**. Horizontal error bars show standard deviation for dose for patients from each dose group, vertical error bars are 68% confidence intervals.

Figures 2 and 3 show incidence data and model predictions for optic neuropathy and retinopathy. Within the dose range bounded by available clinical data, the model predictions are very similar. Notably, for optic neuropathy in patients treated twice daily, curves substantially deviate at doses beyond available clinical data. (Figures 2 and 3)

Table 1 lists the calculated model parameters and confidence intervals. For the considered RION and RIRP data, log-logistic and Poisson-based models consistently yield larger *D_50 _*and smaller *γ *compared to logistic and probit models. In case of optic neuropathy in patients treated twice daily the difference in model parameters is particularly pronounced, albeit with broad confidence intervals due to the small number of events. *D_50 _*is 96.3 Gy in the logistic model and 125.2 Gy in the log-logistic one while *γ *is respectively 1.56 and 0.80.

**Table 1:** Calculated model parameter values and 95% confidence intervals (in parentheses).

Data set	Parameter	Logistic	Log-logistic	Poisson	Probit
RION, twice daily*	D_50_, Gy	96.3 (69.8,∞)	125.2 (71.8, ∞)	119.0 (75.6, ∞)	104.4 (71.7, ∞)
	
	γ	1.56 (0.49,3.18)	0.80 (-0.09,2.38)	0.93(0.42,1.65)	1.27(0.47,2.42)

RION, once daily	D_50_, Gy	94.2 (80.5,146.8)	104.7 (82.8,254.2)	102.0 (83.9,161.9)	96.7 (81.5,150.0)
	
	γ	1.41 (0.84,2.16)	0.88 (0.34,1.60)	0.99 (0.66,1.41)	1.25 (0.78,1.85)

RIRP, twice daily	D_50_, Gy	72.2 (63.9,115.7)	74.2 (63.8,188.7)	75.0 (63.6,133.5)	73.0 (63.8,120.9)
	
	γ	2.16 (0.98,3.97)	1.66 (0.42,3.47)	1.51 (0.72,2.73)	1.91 (0.89,3.43)

RIRP, once daily	D_50_, Gy	72.2 (64.0,91.0)	74.0 (63.1,108.1)	73.0 (62.9,94.4)	72.4 (63.9,91.6)
	
	γ	1.20 (0.73,1.80)	0.84 (0.42,1.40)	0.96 (0.64,1.34)	1.12 (0.71,1.62)

*Abbreviations: RION - radiation-induced optic neuropathy; RIRP - radiation-induced retinopathy.

**Figure 4:**
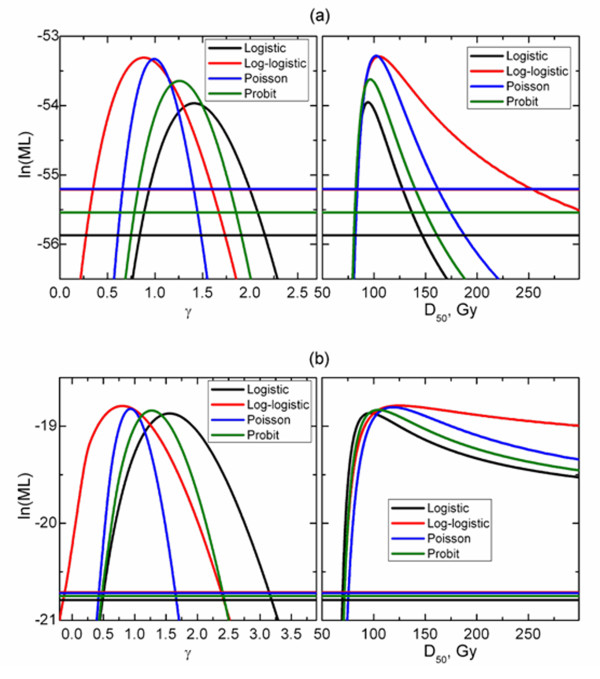
**Log-likelihood function projected onto D_50 _(right panels) and γ (left panels) planes for optic neuropathy in patients treated once daily (a) and twice daily (b)**.

**Figure 5:**
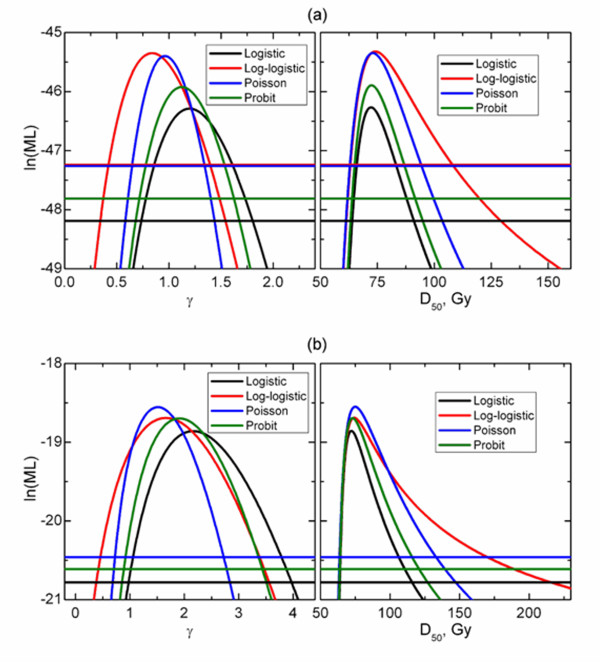
**Log-likelihood function projected onto D_50 _(right panels) and γ (left panels) planes for retinopathy in patients treated once daily (a) and twice daily (b)**.

Figures 4 and 5 show profile likelihood projections on *D_50 _*and *γ *planes as well as model-specific cut-off lines used in derivation of confidence intervals. Similar values of maximum likelihood indicate that different models fit the data equally well. However, not only profiles reach maxima at different *D_50 _*and *γ *values. As shown in Table 1 there is also a substantial difference in calculated confidence intervals. (Figures 4 and 5)

The sensitivity of model parameters to the used α/β value were assessed. When α/β = 1 Gy was used to convert *D_min _*to NTD values of the model parameters, *D_50 _*and *γ *for RION in patients treated once daily were 96.0 Gy and 1.34, 107.6 Gy and 0.82, 103.9 and 0.95, and 98.1 Gy and 1.20 for the logistic, log-logistic, Poisson-based and probit models, respectively. For α/β = 5 Gy, corresponding values in the same order were 92.2 Gy and 1.52, 101.2 Gy and 0.98, 100.0 Gy and 1.05, and 94.6 Gy and 1.34. For RION in patients treated twice daily and α/β = 1 Gy, parameter values were 91.0 Gy and 1.52, 120.9 Gy and 0.76, 113.1 Gy and 0.91, and 98.8 Gy and 1.24 for the logistic, log-logistic, Poisson-based and probit models, respectively. After α/β was set to 5 Gy, the corresponding values were 106.9 Gy and 1.61, 135.8 Gy and 0.85, 132.3 Gy and 0.95, and 116.4 Gy and 1.30. Parameter values obtained for α/β = 1 Gy and 5 Gy envelop values shown in Table 1. Sensitivity to α/β was modest.

## Discussion

Despite astute observations by Bentzen and Tucker, 1997, showing that the slope of TCP dose-response is model-dependent, even if fitting was performed to the same data, dependence of the model parameters on the choice of the model is generally not appreciated. Limited attention has also been devoted to demonstrating conflicts in plan ranking or in predicting consequences of dose boosting in partial volumes between common models [28-30]. In this report, the lingering question to what extent model predictions are model dependent has been studied in a systematic manner. As expected no model can be deemed a preferred model and all four models agree well within the range of the clinical data. Dosimetric parameters of clinical relevance, for example NTCP at 55 and 60 Gy, doses typically used as constraints in IMRT planning [31], would therefore be model-independent as long as there is incidence data in this dose range. These NTCP differences were in fact < 1% for RION and < 3% for RIRP, see Figures 2 and 3. The same applied to *D_5 _*and *D_10_*, doses corresponding to 5 and 10% incidence of complications. Figures 2 and 3 show that the differences in these values predicted by different models were < 1.5 Gy for RION and < 4.5 Gy for RIRP.

However, for the RION data set for patients treated twice daily, where incidence data covered the smallest in range of the four sets, predictions beyond the range of data availability became quite model dependent. Not only is this reflected in large discrepancies in *D_50 _*values; *D_20_*, dose corresponding to 20% incidence of RION, is 74.9 Gy for the logistic model. This contrasts with 81.2 Gy calculated from the log-logistic model. This would be consequential for dose escalation protocols relying on extrapolated incidence of complications.

The trend that the log-logistic and Poisson-based models yielded larger *D_50 _*and smaller *γ *compared to logistic and probit models was observed. This is likely related to the shape of the dose-response characteristic of a specific model as well as the limited range of incidence of complications. While this ideally has to be proven mathematically, we can speculate that the trend is driven by differences in model predictions in the incidence range of concern for this study. Figure 1 shows that the log-logistic and Poisson-based models reach complication probabilities of the order of 10-20% at doses larger than the logistic and probit models. In Figure 1, models were matched according to *D_50 _*and *γ*. One can speculate that if models were forced to overlap in the range of clinically observed incidences of complications, i.e., < 20%, larger *D_50 _*would be expected for the log-logistic and Poisson-based models.

The model dependence is typically not specifically addressed in literature reviews that present compilations of model parameters [32]. It is conceivable that the large *D_50 _*values reported for xerostomia by Munter *et al*. 2004 and Munter *et al*. 2007 were at least partly due to their choice of the log-logistic model. In this regard, generic statements based on shallow dose-response of *γ*≤1 should be made with caution as well. As shown in this study a difference on the order of factor of two has been observed for the RION data set for patients treated twice daily (*γ *= 0.8 and 1.56, Table 1). This data set was limited in complication incidence. Even for the RIRP data set covering a broad range of incidence, substantial variations in *γ *were seen while variations in *D_50 _*were minor. Disagreement in model parameters cannot be viewed solely as a reflection of differences in underlying data. While this conclusion would be valid for data sets covering a broad range of incidences, human data for a good reason is typically limited to low incidences of complication. It has to be stated that while the log-logistic model predicted shallow dose-response, the only way to claim inferiority of this model is to demonstrate that its predictions contradict clinical data. The model cannot be disregarded based on how plausible its parameters and predictions to larger doses may appear compared to other models. It is unfortunate that publications showing model predictions often do not also show clinical data in the same plot, as shown in Figures 2 and 3. This provides readers with a better understanding of the spread of clinical data in dose, incidence of toxicity and statistical uncertainty.

Variations in confidence intervals were substantial. This at least in part can be connected with model parameters themselves. In particular, log-logistic model yielded the larger *D_50 _*as well as broader upper limit for *D_50_*. Having said that, for RIRP data sets, *D_50 _*were consistent between the models and still upper confidence interval was by far the largest for the log-logistic model. The reverse argument applies to *γ*, log-logistic model providing the broadest lower limit. Confidence intervals calculated for model parameters were broad, which relates to the small number of events. In particular, patients treated twice daily showed a low incidence of complications. Consequently, model parameters can be only estimated with substantial uncertainties. While this precludes being definitive in comparing model behavior, this is a common problem in testing model predictions. The presented analysis therefore is representative of a practical situation of dose-response analysis and use of model parameters.

The maximum likelihood method was used in this study to estimate model parameters. It should be noted that the choice of the method may impact parameter values and confidence intervals. Bentzen and Tucker [16], 1997, analyzed dose-response for control of neck nodes. The authors showed that the *D_50 _*value was not sensitive to whether the maximum-likelihood or least-squares method was used to estimate parameters of the logistic model. Least squares, however, led to a substantially narrower confidence interval. Also, a significant difference in *γ *was seen. This potentially adds to uncertainties associated with comparing model parameters reported by various authors.

In this study the analysis was restricted to dose-response rather than dose-volume response. The way volume effect is handled by different models will have an impact on obtained model parameters. Commonly, dose-volume-response models have a designated parameter describing the strength of volume dependence. However, models designed to describe the incidence of complications in serial organs may not require this parameter [12]. Furthermore, the slope of dose-response may or may not be volume-dependent. This leads to differences in model parameters. However, the preferred model often cannot be established because of the uncertainties in clinical data.

Venturing in dose range not covered by clinical data is unavoidable in biologically-guided IMRT optimization. This makes the choice of the model critical. Presently the choice of NTCP models is driven by personal preferences, availability of software and historical reasons. A practice of selecting a model and "calibrating" the model to make it consistent with locally seen outcomes is encouraged [8]. When advanced biologically-driven treatment planning is used, e.g., to account for biological properties of tumors and normal tissues [5] or effect of geometric errors [33] there has to be an understanding that a choice of the model would dictate the penalty.

The results of IMRT optimization, including biologically-driven optimization, are of course subject to assessing the plan for its clinical suitability. If the plan is deemed clinically unsuitable, optimization can be re-run and navigated towards the desired result by changing weighting factors. Therefore, differences in model predictions can be offset in biologically-based optimization unless absolute values are used. A similar argument applies to plan ranking. The model does not have to be quantitatively accurate as long as it ranks a radiobiologically more desirable plan higher than less desirable. Use of biological models for plan ranking cannot be separated from DVH handling. If NTCP is calculated following a DVH reduction using an independent method, e.g., using power-law-based EUD [13], then plan ranking based on EUD is sufficient. Further, calculation of NTCP becomes redundant. If, however, NTCP is calculated directly from the DVH or a popular effective volume DVH reduction method is used [34], ranking would be based on calculated NTCP. It has been shown, however, that plan ranking can be model-dependent [30]. Quantitative use of biological models to predict complication rates for a proposed clinical trial or treatment schedule may depend on the choice of the model. Commonly, approaches based on changing fractionation to maintain the rate of complications but to improve local control are used. Also, RT protocols based on individualized prescription with an intent to keep NTCP below a pre-set level have been advocated and used clinically [35]. These approaches indirectly validate model predictions; however, their clinical implementation has to have clearly stated rules for what would be regarded as excess toxicity.

## Conclusions

Based on the analysis of radiation-induced optic neuropathy and retinopathy data, we conclude that large variations in model parameters may be observed between the models if data are restricted in incidence range. This leads to inconsistencies in model projections. For the considered data sets the log-logistic model tends to lead to larger *D_50 _*and lower *γ *compared to other models. This, however, does not constitute reasons for claiming inferiority of this model. This claim can be only made based on a comparison of model predictions and clinical data. Statements regarding inconsistencies between data sets from different institutions should not be based solely on reported model parameters as the latter are model-dependent.

## Competing interests

The authors declare that they have no competing interests.

## Authors' contributions

WS and NB conceived the idea and VM, WS, and NB designed the study. NB collected the data. VM, LM, and WS performed the analysis. VM drafted the manuscript with the help of WS, LM, and NB. All authors read and approved the final manuscript.
